# Proprietary Remedies in Veterinary Practice

**Published:** 1895-07

**Authors:** E. Wallis Hoare

**Affiliations:** F.R.C.V.S. (Cork)


### PROPRIETARY REMEDIES IN VETERINARY PRACTICE.

IN the present scientific age it would naturally be imagined that every remedy or medicinal agent employed in the treatment of disease should have its precise action perfectly familiar to the prescriber.

He should be aware of the indications for the selection of the special medicinal agent, and should possess a clear perception of the manner in which therapeutic results are to be brought about.

This being the case, it is apparent that the scientific practitioner will not prescribe any agent or combination of agents the composition and actions of which are unknown to him.

It is not necessary, however, that he should be acquainted with the exact chemical preparation of the medicinal agent, or of its convenient form and combinations. For example, take that useful and convenient preparation known as chlorodyne; we are aware that it contains a certain proportion of morphine to the ounce, also of spirits of chloroform, and any other carminative that we may desire to add.

This we would not class as a proprietary medicine, because we are aware of its contents, although various wholesale chemists may claim special virtues for their preparations of the compound. Again, take that excellent antiseptic known as creolin; we are aware of its derivation, although we are not acquainted with the chemical processes necessary to prepare it; but from practical experience in its use we know its value as an antiseptic, both internally and externally, and its superiority to the time-honored carbolic acid.

But there are remedies advertised, with long-sounding titles, concerning which we are utterly ignorant of the composition or of the physiological actions, and it is with feelings of surprise that we note testimonials from practitioners in favor of the medicinal virtues of these preparations.

Still greater should be our surprise to find that patent preparations publicly advertised contain testimonials from qualified veterinary surgeons.

It was only lately that I saw some of these testimonials in a pamphlet issued by a certain firm, while in the same production was to be found in many places illustrations of cases in which one and two "vets" failed to cure; but the preparations advertised brought about a complete recovery. Surely it is high time that practitioners should cease to employ or recommend patent remedies.

The Pharmacopœia contains sufficient medicinal agents to select from without descending to adopt the nostrums of veterinary chemists.

But it certainly appears strange that the professional journals should contain advertisements of these empirical compounds, and we read at present an advertisement of a celebrated French "Red Ointment," which is "the only known substitute for the hot iron;" also of a "Black Mixture," which is a "specific for broken-kneed horses;" and other abominable announcements of a similar nature, finishing up with the insulting sentence, "Customary allowances to veterinary surgeons."

Such advertisements should not be inserted in journals which claim to represent scientific veterinary medicine and surgery, and it is only to be hoped that these have got no lay readers.

We all know to our cost the enormous amount of injury done to practitioners by the employment of patent preparations. There is an innate desire in the minds of all horse- and stock-owners for patent embrocations, red drenches, mange cures, etc., and they will allow these empirical agents a far longer time to produce effects than they will permit professional attendance; and, as for expense, this is no object so long as the preparation has a high-flowing name, a good color, and a characteristic odor.

In country districts especially, the amount of patent cure-alls sold is enormous, and it frequently happens that these are freely tried before professional assistance is sought.

Then we have dispensing chemists advertising and selling all forms of veterinary medicines, thus taking away the legitimate profits of the practitioner.

Professional fees in a country district, exclusive of medicines, are not by any means proportionate to the work done and expected; hence, it behooves us to protect our interests by supplying our own medicines and endeavoring to convert the tastes of our clients from patent preparations.

No doubt, it is sometimes refreshing to be told that we prescribe far too much medicine, and that we should depend more on hygiene, etc., in practice; this fact we must admit in many instances, but we fear that the millennium of professional progress must be reached before we can impress the correctness of this theory sufficiently on the owners and attendants of animals.—E. WALLIS HOARE, F.R.C.V.S. (Cork), *in The Veterinary Record*, June, 1895.

